# Use of statins or NSAIDs and survival of patients with high-grade glioma

**DOI:** 10.1371/journal.pone.0207858

**Published:** 2018-12-03

**Authors:** Corinna Seliger, Julia Schaertl, Michael Gerken, Christian Luber, Martin Proescholdt, Markus J. Riemenschneider, Michael F. Leitzmann, Peter Hau, Monika Klinkhammer-Schalke

**Affiliations:** 1 Department of Neurology, Regensburg University Medical Center, Regensburg, Germany; 2 Wilhelm Sander-NeuroOncology Unit, Regensburg University Medical Center, Regensburg, Germany; 3 Tumor Center, Institute for Quality Assurance and Health Services Research, University of Regensburg, Regensburg, Germany; 4 Department of Neurosurgery, Regensburg University Medical Center, Regensburg, Germany; 5 Department of Neuropathology, Regensburg University Hospital, Regensburg, Germany; 6 Department of Epidemiology and Preventive Medicine, University of Regensburg, Regensburg, Germany; University of Portsmouth, UNITED KINGDOM

## Abstract

**Background:**

High-grade glioma (HGG) is associated with a limited prognosis. Drug repurposing has become of increasing interest to improve standard therapy. Statins and NSAIDs inhibit glioma cell growth *in vitro* and *in vivo*, but data on statin and NSAID treatment in relation to survival of patients with HGG are sparse.

**Methods:**

We performed multivariable adjusted Cox-regression analyses among 1,093 patients with HGG from a regional cancer registry to obtain Hazard Ratios (HRs) with 95% Confidence Intervals (CIs) for overall survival (OS) and progression-free survival (PFS) according to treatment with statins or NSAIDs. Data on dose and duration of treatment was mostly lacking in our analysis, therefore we were not able to perform dose-response analyses.

**Results:**

Use of statins was unrelated to OS or PFS of glioma patients. Use of aspirin was associated with prolonged OS and PFS in patients with WHO grade III, but not WHO grade IV glioma. Use of other NSAIDs (diclofenac, ibuprofen) or non-NSAID analgesics (paracetamol) was mostly unrelated to survival of glioma patients. Use of selective COX-2 inhibitors and metamizol was related to inferior patient survival in parts of the analyses.

**Conclusions:**

Use of statins or NSAIDS, including aspirin, was not associated with prolonged OS or PFS of patients with WHO grade IV glioma in our selected cohort. There was an indication for improved survival in patients with WHO grade III glioma using aspirin, but further studies are needed to confirm our first observation.

## Introduction

High-grade gliomas are amongst the deadliest of all cancers [1]. They are classified into World Health Organization (WHO) grades III and IV, with isocitrate dehydrogenase mutation (IDHmut) or IDH wildtype (IDHwt) status. Tumors with presence of an IDH mutation and concurrent loss of heterozygosity (LOH) of chromosome arms 1p and 19q are designated as oligodendrogliomas [2]. Factors influencing survival of patients with HGG include age at diagnosis, extent of resection, clinical performance score, *MGMT* promoter methylation status, primary therapy and presence of relevant comorbidities [3–5].

Drug repurposing has evolved as a promising field in neurooncology [6]. Several biological mechanisms exist, through which commonly used medications such as statins or NSAIDS may influence glioma survival, including targeting of the mevalonate [7–14] or cyclooxygenase pathways [15–26].

Prior studies are inconclusive with both improved [27], but also unchanged survival of glioblastoma patients [28, 29] after statin use. Also, use of NSAIDs and specifically use of selective COX-2 inhibitors has shown modest effectiveness in some metronomic schemata for glioblastoma [30–33], but not in others [34–36].

Based on possible biological mechanisms and in consideration of the low number and inconclusive results of prior studies investigating survival of patients with HGG after treatment with statins or NSAIDS, we performed this large retrospective cohort study.

## Patients and methods

### Data source and study population

We used the population-based clinical cancer registry Regensburg to obtain data from all patients diagnosed with WHO grade III and IV glioma in the region of Lower Bavaria and Upper Palatinate according to the ICD-10 and ICD-0 classification between January 1, 1998 and December 31, 2013. The area has about 2.1 million inhabitants, 53 regional hospitals, a university hospital and over 1,500 practitioners. According to estimates of the German Robert-Koch Institute (RKI) 98% of all cancer cases are recorded in the cancer registry [37]. The cancer registry routinely assesses sex, age at diagnosis, year of diagnosis, primary therapy, status of molecular markers (*MGMT* promoter methylation status; *IDH* mutational status, both implemented since 2009), date of first progression, date of last follow-up, and date of death. Vital status of the patient cohort was also verified by death certificates and information from population registries.

*IDH* mutational and *MGMT* promotor methylation status were determined as described [38].

Patients with other cancers (previously or concurrently, except non-melanoma skin cancer), patients with missing follow-up data, and patients younger than 18 years were excluded. The study was performed in accordance with the Declaration of Helsinki (data collection and analysis was anonymous), and was approved by the Bavarian Law of Cancer Registration.

### Exposures

Information on the extent of resection (biopsy, complete resection, partial resection, unknown), Karnofsky Performance Score (KPS; 100, 80–90, 60–70, 40–50, 10–30, unknown), body mass index (BMI; <25, 25–29.9, 30–34.9, ≥35 kg/m2, unknown), comorbidities (including hyperlipidemia and cardiac insufficiency), use of co-medications (including statins (yes, no; namely simvastatin, atorvastatin, cerivastatin, fluvastatin, lovastatin, pravastatin); NSAIDS: diclofenac (yes, no), ibuprofen (yes, no), selective COX-2 inhibitors (yes, no; namely celecoxib, rofecoxib and etoricoxib); non-NSAID analgesics: metamizol (yes, no), or paracetamol (yes, no)) was collected by scanning patient discharge letters, which are collected in the cancer registry. If data were lacking in the registry, we additionally sent standardized questionnaires to general practitioners. We had complete data for all 1,093 patients (among others) on the date of diagnosis, age at diagnosis, WHO grade, sex, primary therapy and use of medications (yes/no). For the MGMT-methylation status, Karnofsky Performance Score, extent of resection and body-mass index we had lacking data as specified in Table 1. The response rate to the questionnaires was 21%. For about 30% of patients we had information on dose and duration of used co-medications.

**Table 1:** Baseline characteristics according to statin use.

		Statin use					
		Yes (122, 11.2%)		No (971, 88.8%)		Total (1,093, 100%)	
		count	%	count	%	count	%
Sex	Male	66	54.1%	553	57.0%	619	56.6%
	Female	56	45.9%	418	43.0%	474	43.4%
Age at diagnosis	< 50	15	12.3%	268	28.6%	283	26.2%
	50–59	30	24.6%	230	23.7%	260	23.8%
	60–69	39	32.0%	261	26.9%	300	27.4%
	> 70	38	31.1%	212	21.8%	250	22.9%
Year of diagnosis	1998–2001	14	11.5%	165	17.0%	179	16.4%
	2002–2005	22	18.0%	271	27.9%	293	26.8%
	2006–2009	20	16.4%	208	21.4%	228	20.9%
	2010–2013	66	54.1%	327	33.7%	393	36.0%
WHO grade	III	16	13.1%	215	22.1%	231	21.1%
	IV	106	86.9%	756	77.9%	862	78.9%
MGMT-Promotor-Methylation	Methylation	28	23.0%	112	11.5%	140	12.8%
	Wildtyp	21	17.2%	124	12.8%	145	13.3%
	ns	73	59.8%	735	75.7%	808	73.9%
IDH1	Mutation	5	4.1%	49	5.0%	54	4.9%
	Wild type	32	26.2%	146	15.0%	178	16.3%
	ns	85	69.7%	776	79.9%	861	78.8%
Karnofsky-Performance Score (class. ECOG)	100 ECOG 0	12	9.8%	128	13.2%	140	12.8%
	80–90 ECOG 1	41	33.6%	260	26.8%	301	27.5%
	60–70 ECOG 2	31	25.4%	132	13.6%	163	14.9%
	< 50 ECOG 3, 4	12	9.8%	68	7.0%	80	7.3%
	ns	26	21.3%	383	39.4%	409	37.4%
Primary therapy	OP+Rad+Chemo	62	50.8%	429	44.2%	491	44.9%
	OP+Rad	12	9.8%	154	15.9%	166	15.2%
	OP	11	9.0%	91	9.4%	102	9.3%
	Rad+Chemo	15	12.3%	80	8.2%	95	8.7%
	supportive/others	22	18.1%	217	22.4%	239	22.0%
Extent of resection	complete	3	2.5%	36	3.7%	39	3.6%
	incomplete	26	21.3%	118	12.2%	144	13.2%
	biopsy	19	15.6%	35	3.6%	54	4.9%
	ns	74	60.7%	782	80.5%	856	78.3%
BMI	< 25.0	18	14.8%	173	17.8%	191	17.5%
	25.0–29.9	32	26.2%	158	16.3%	190	17.4%
	30+	25	20.5%	91	9.4%	116	10.6%
	ns	47	38.5%	549	56.5%	596	54.5%
Total		122	100.0%	971	100.0%	1093	100.0%

ns = not specified

### Statistical analysis

We first analysed factors possibly related to glioma survival using Kaplan-Meier estimates. In our main analysis, we conducted multivariable COX regression with forward selection to obtain Hazard Ratios (HRs) with 95% Confidence Intervals (CIs) for overall and progression-free survival of patients with HGG according to treatment with statins, NSAIDs or non-NSAID analgesics. As potentially confounding variables we included WHO grade of glioma, sex, age at diagnosis, year of diagnosis, BMI, *MGMT* promoter methylation status, *IDH* mutational status, Karnofsky Performance Score, extent of resection and primary therapy. We included missing values in a separate category in the multivariable regression model. In addition, we also performed a minimal model only including age, sex and WHO grade of glioma to prevent bias due to multicollinearity and statistical over-control. Also, medications were investigated in separate models.

We set the type I error at 5% for all statistical analyses and all tests were two-tailed. Analyses were performed using SPSS statistical software version 23. We performed subanalyses investigating drug use stratified by WHO grade of glioma, taking into consideration that the analyses for WHO grade III are mostly underpowered.

## Results

We ascertained 1,093 patients with HGG in our database. Of these, slightly less than half (43.4%) were women. 862 patients were diagnosed as WHO grade IV, 231 patients as WHO grade III glioma. The mean age of HGG patients was 59 (± 13.8) years.

Patient characteristics for all HGG patients and according to statin use are displayed in Table 1, according to aspirin use in Table 2 and according to use of diclofenac, ibuprofen, selective COX-2 inhibitors, metamizol and paracetamol in S1–S5 Tables. Median follow-up was 7.3 years. Among patients with known dose, duration and indication of aspirin use (22 patients, 31.4% of all HGG patients taking aspirin) 68% of patients were taking aspirin before the diagnosis of glioma. 91% of patients took aspirin at a dose of 100 mg daily for cardiovascular diseases as continuous treatment. Among patients with more detailed information on statin use (17 patients, 13,9% of all patients taking statins), 94.1% of patients took statins as continuous treatment with a median dose of 20 mg/day. 84.6% of patients were taking statins before the diagnosis of glioma. 50% of patients on statins had a coding for hyperlipidemia, in addition statins were used for cardiovascular disease.

**Table 2:** Baseline characteristics according to aspirin use.

		Aspirin use					
		Yes (69, 6.3%)		No (1,024, 93.7)		Total (1,093, 100%)	
		count	%	count	%	count	%
Sex	Male	45	65.2%	574	56.1%	619	56.6%
	Female	24	34.8%	450	43.9%	474	43.4%
Age at diagnosis	< 60	21	30.4%	522	50.9%	543	49.7%
	60–69	15	21.7%	285	27.8%	300	27.4%
	> 70	33	47.8%	217	21.2%	250	22.9%
Year of diagnosis	1998–2005	20	29.0%	452	44.2%	472	43.2%
	2006–2013	49	71.0%	572	55.9%	621	56.9%
WHO grade	III	11	15.9%	220	21.5%	231	21.1%
	IV	58	84.1%	804	78.5%	862	78.9%
MGMT-Promotor-Methylation	Methylation	16	23.2%	124	12.1%	140	12.8%
	Wildtyp	15	21.7%	130	12.7%	145	13.3%
	ns	38	55.1%	770	75.2%	808	73.9%
IDH1	Mutation	2	2.9%	52	5.1%	54	4.9%
	Wild type	20	29.0%	158	15.4%	178	16.3%
	ns	47	68.1%	814	79.5%	861	78.8%
Karnofsky-Performance Score (class. ECOG)	> 90 ECOG 0,1	30	43.5%	411	40.1%	441	40.3%
	< 70 ECOG 2, 3, 4	26	37.7%	217	21.2%	243	22.2%
	ns	13	18.8%	396	38.7%	409	37.4%
Primary therapy	OP+Rad+Chemo	34	49.3%	457	44.6%	491	44.9%
	OP+Rad	13	18.8%	153	14.9%	166	15.2%
	supportive/others	22	31.9%	414	40.5%	436	39.9%
Extent of resection	complete	4	5.8%	35	3.4%	39	3.6%
	incomplete	15	21.7%	129	12.6%	144	13.2%
	biopsy	8	11.6%	46	4.5%	54	4.9%
	ns	42	60.9%	814	79.5%	856	78.3%
BMI	< 25.0	14	20.3%	177	17.3%	191	17.5%
	25.0–29.9	16	23.2%	174	17.0%	190	17.4%
	30+	15	21.7%	101	9.9%	116	10.6%
	ns	24	34.8%	572	55.9%	596	54.5%
Total		69	100.0%	1024	100.0%	1093	100.0%

Limited information on non-aspirin NSAIDs revealed that they were mostly prescribed non-continuously and after the diagnosis of glioma (first NSAID prescription after glioma diagnosis in 85% (ibuprofen), 37.5% (diclofenac) or 100% (COX-2 inhibitors, metamizol and paracetamol).

In Kaplan-Meier survival analyses worse overall survival was observed in patients with increasing age, WHO grade IV glioma, lower Karnofsky Performance Score, incomplete resection or biopsy only, unmethylated *MGMT* promoter status, absence of *IDH* mutation and absence of combined radiochemotherapy.

A diagnosis of hyperlipidemia (HR for OS = 0.96; 95%CI = 0.78–1.18; p-value = 0.686, HR for PFS = 0.99; 95%CI = 0.81–1.21; p-value = 0.922), cardiac insufficiency (HR for OS = 1.27; 95%CI = 0.88–1.83; p-value = 0.201, HR for PFS = 1.32; 95%CI = 0.92–1.89; p-value = 0.127) or myocardial infarction (HR for OS = 1.15; 95%CI = 0.73–1.82; p-value = 0.535, HR for PFS = 0.98; 95%CI = 0.63–1.52; p-value = 0.927) was unrelated to overall or progression-free survival of patients with HGG. There was a borderline significant positive association between a history of stroke and survival of patients with HGG (HR for OS = 0.72; 95%CI = 0.52–1.01; p-value = 0.054, HR for PFS = 0.77; 95%CI = 0.56–1.06; p-value = 0.111).

**Fig 1:**
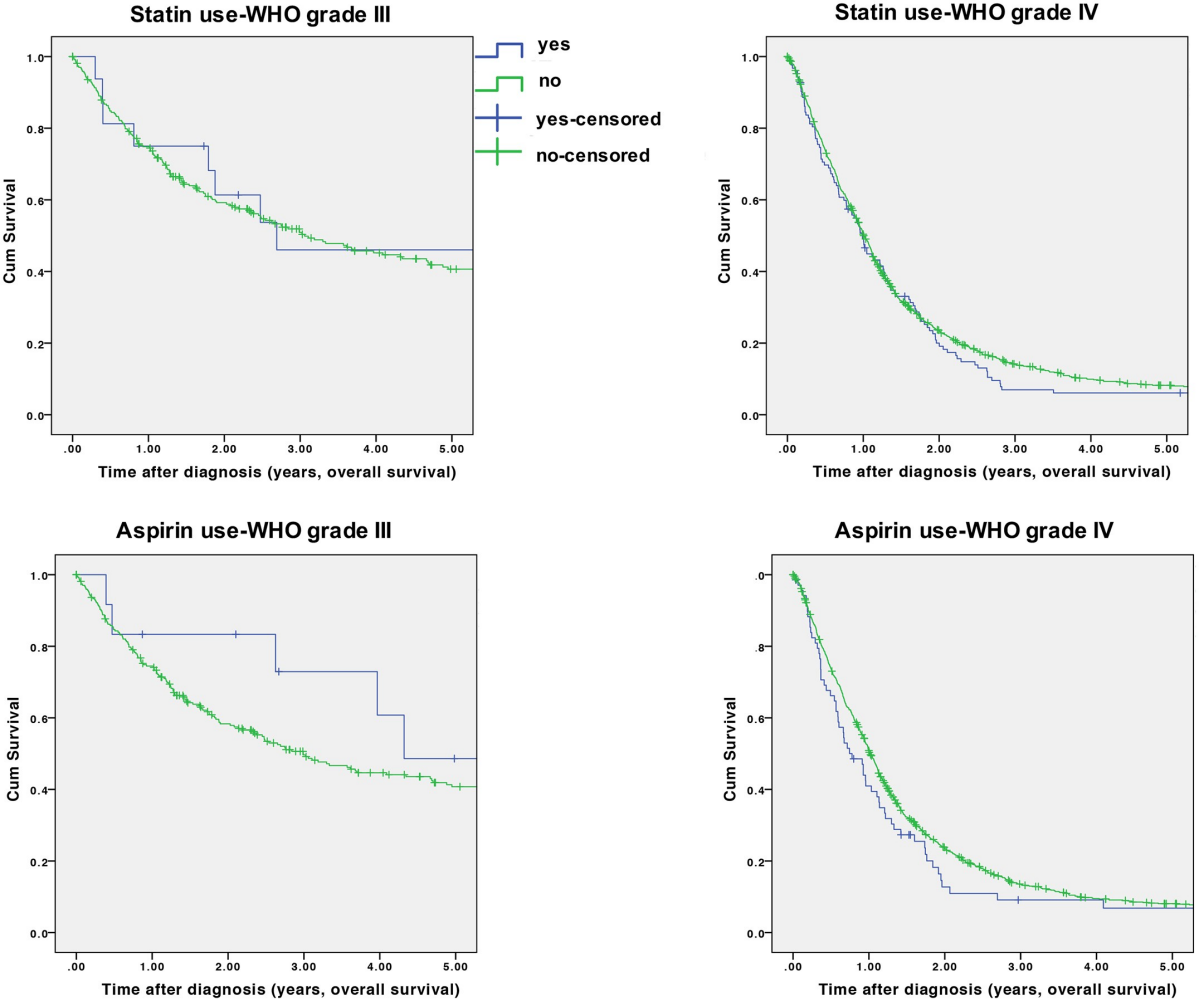
Kaplan-Meier survival curves for WHO grade III or IV glioma according to use of statins or aspirin.

Use of statins was unrelated to overall or progression-free survival of HGG patients (HR for OS = 0.95; 95%CI = 0.77–1.18, HR for PFS = 0.91; 95%CI = 0.74–1.13), also when stratified by WHO grade of glioma (Fig 1). When excluding patients with cardiovascular disease, the results for statin use and OS remained comparable to those of our main analysis, whereas the results for PFS were moved towards a non-significant survival-prolonging association of statin use with progression-free survival (HR for OS = 0.97; 95%CI = 0.78–1.21; HR for PFS = 0.87; 95%CI = 0.70–1.08). In contrast, use of aspirin was related to significantly better overall and progression-free survival in patients with WHO grade III (HR for OS = 0.25; 95%CI = 0.10–0.63, HR for PFS = 0.31; 95%CI = 0.14–0.72), but not WHO grade IV glioma (HR for OS = 1.02; 95%CI = 0.76–1.58, HR for PFS = 1.04; 95%CI = 0.77–1.39, Fig 1). No relations with overall or progression-free-survival were noted for use of diclofenac, ibuprofen or paracetamol. In contrast, use of selective COX-2 inhibitors was associated with a slightly worse PFS of glioma patients (HR for PFS = 1.43; 95%CI = 1.05–1.96) and metamizol was associated with a significantly worse PFS and borderline significantly worse OS in patients with WHO grade III glioma (Table 3).

**Table 3:** Survival of HGG patients in relation to statin, aspirin or non-aspirin-NSAID use.

Variable	Number of cases (%)	Adjusted HR* (95% CI)	p-value
High-grade glioma	n = 1,093		
Overall Survival			
Statins	122 (11.2)	0.95 (0.77–1.18)	0.641
Aspirin	69 (6.3)	0.71 (0.53–0.94)	0.016
Diclofenac	18 (1.6)	1.03 (0.63–1.70)	0.907
Ibuprofen	43 (2.4)	1.30 (0.93–1.83)	0.123
Selective COX-2 inhibitors	50 (4.6)	1.06 (0.75–1.48)	0.752
Metamizol	55 (5.0)	1.00 (0.73–1.38)	0.983
Paracetamol	13 (1.2)	0.83 (0.42–1.63)	0.588
Progression-free Survival			
Statins	122 (11.2)	0.91 (0.74–1.13)	0.406
Aspirin	69 (6.3)	0.80 (0.61–1.05)	0.104
Diclofenac	18 (1.6)	1.00 (0.62–1.64)	0.986
Ibuprofen	43 (2.4)	1.26 (0.91–1.74)	0.165
Selective COX-2 inhibitors	50 (4.6)	1.43 (1.05–1.96)	0.026
Metamizol	55 (5.0)	0.99 (0.73–1.36)	0.957
Paracetamol	13 (1.2)	0.95 (0.52–1.76)	0.876
WHO grade III glioma	n = 231		
Overall Survival			
Statins	16 (6.4)	1.16 (0.60–2.26)	0.657
Aspirin	11 (4.8)	0.25 (0.10–0.63)	0.003
Diclofenac	2 (0.9)	1.16 (0.28–4.79)	0.838
Ibuprofen	8 (3.5)	2.14 (0.92–4.95)	0.076
Selective COX-2 inhibitors	11 (4.8)	1.56 (0.67–3.62)	0.304
Metamizol	10 (4.3)	2.08 (0.94–4.58)	0.070
Paracetamol	3 (1.3)	0.82 (0.11–6.02)	0.848
Progression-free Survival			
Statins	16 (6.4)	1.06 (0.55–2.06)	0.857
Aspirin	11 (4.8)	0.31 (0.14–0.72)	0.007
Diclofenac	2 (0.9)	0.91 (0.22–3.77)	0.901
Ibuprofen	8 (3.5)	1.86 (0.81–4.28)	0.147
Selective COX-2 inhibitors	11 (4.8)	1.67 (0.78–3.56)	0.185
Metamizol	10 (4.3)	2.46 (1.17–5.17)	0.017
Paracetamol	3 (1.3)	0.85 (0.12–6.23)	0.874
WHO grade IV glioma	n = 862		
Overall Survival			
Statins	44 (5.1)	0.96 (0.76–1.20)	0.714
Aspirin	58 (6.7)	1.02 (0.76–1.58)	0.886
Diclofenac	16 (1.9)	0.98 (0.57–1.67)	0.939
Ibuprofen	35 (4.1)	1.21 (0.83–1.75)	0.323
Selective COX-2 inhibitors	39 (4.5)	0.98 (0.68–1.42)	0.926
Metamizol	45 (5.2)	0.92 (0.65–1.30)	0.628
Paracetamol	10 (1.2)	0.83 (0.40–1.69)	0.602
Progression-free Survival			
Statins	44 (5.1)	0.91 (0.72–1.14)	0.396
Aspirin	58 (6.7)	1.04 (0.77–1.39)	0.805
Diclofenac	16 (1.9)	1.03 (0.61–1.74)	0.911
Ibuprofen	35 (4.1)	1.28 (0.91–1.82)	0.162
Selective COX-2 inhibitors	39 (4.5)	1.31 (0.93–1.86)	0.123
Metamizol	45 (5.2)	0.85 (0.61–1.20)	0.360
Paracetamol	10 (1.2)	0.95 (0.50–1.81)	0.877

* Model adjusted for age at diagnosis, sex, year of diagnosis, WHO grade (only in complete study), BMI, Karnofsky Performance Score, Extent of resection, MGMT promoter methylation status, IDH mutation status, primary therapy

As a minimal model, we repeated analyses on OS of HGG patients only adjusting for age, gender and WHO grade of glioma with no relevant changes to our main results (HR for aspirin = 0.71; 95%CI = 0.54–0.93; HR for ibuprofen = 1.24; 95%CI = 0.89–1.71; HR for statins = 0.93; 95%CI = 0.76–1.14; HR for diclofenac = 1.10; 95%CI = 0.67–1.81; HR for COX-2 inhibitors = 0.81; 95%CI = 0.59–1.11).

## Discussion

In this large retrospective cohort study, we observed that aspirin use was related to significantly improved overall and progression-free survival of patients with WHO grade III glioma, although the analyses are likely underpowered. In contrast, no significant relations were noted for use of aspirin in patients with statins, non-aspirin NSAIDs, non-NSAID analgesics and aspirin in patients with WHO grade IV glioma.

Statin use has been found to be related to reduced cancer-related mortality for a variety of cancers [39], but data on brain cancer and specifically on glioma is sparse. In experimental studies, statins were found to inhibit cell proliferation and migration and to induce apoptosis [8, 10, 12–14, 40–43]. Molecular mechanisms include inhibition of the mevalonate metabolism with downstream modulation of the Ras–Raf–MEK–ERK signaling pathway [7] or Akt signaling [12]. In line with those findings, one study based on 339 glioblastoma patients from the Danish Cancer registry found a reduced HR of death among patients with prediagnostic statin use (HR = 0.79; 95% CI: 0.63–1.00) [27]. However, a large Danish study of 5,245 patients reported no significant association with death from brain cancer among statin users (HR = 0.95; 95%CI = 0.81–1.12) [39]. In a pooled analysis of randomized clinical trials, including 810 patients, there was no association between statin use and survival of patients with primary glioblastoma [29]. Further, in a study performed in Texas, preoperative statin use was not associated with improved survival among 284 patients with glioblastoma (HR for PFS = 0.94, 95% CI = 0.70–1.26) [28]. There have been some recent recommendations in statin epidemiology literature that some analyses (meta-analyses) should seriously consider excluding patients with cardiovascular diseases as a best practice [44]. We therefore performed an additional analysis excluding patients with stroke, cardiac insufficiency or a history of myocardial infarction what did not change our results for OS, but led to a borderline survival-prolonging association of statin use and PFS in patients with HGG.

Our study differs from previous studies in that we included the by far largest number of patients with high-grade glioma (1,093 patients) and also performed analyses specifically for WHO grade III gliomas. In addition, our study included statistical adjustments for molecular marker status as well as primary therapy, KPS, BMI and extent of resection, factors that are well known to influence survival of patients with HGG.

Non-steroidal inflammatory drugs act by inhibition of cyclooxygenase (COX-1 or 2). High expression of COX-2 has been linked to poor survival of glioma patients in multivariate adjusted analyses [45]. Inhibition of prostanoid synthesis by NSAIDs leads to a blockage of immunosuppressive lymphoid and myeloid cells within the HGG tumor microenvironment [46, 47]. Inhibition of COX-2 has therefore prompted interest as possible adjuvant treatment for glioma and was implemented in several metronomic schemata for the treatment of glioma, with modest positive effects in some studies [30–33], but not in others [34–36] mainly investigating selective COX-2 inhibitors. In studies evaluating the risk of glioma among NSAID users, several studies reported an inverse association between NSAID use and glioma risk [48, 49], but others reported null associations or non-significant inverse associations between use of aspirin or non-aspirin NSAIDs and glioma risk [50, 51]. Interestingly, one study stratified according to type of NSAID and noted that only aspirin use was significantly associated with reduced glioma risk [52] and another study showed overall null results overall but found a suggestive reduction in glioma risk only in patients with long-term use of aspirin (OR = 0.80; 95%CI = 0.53–1.21) [53]. Aspirin is the only NSAID that leads to an irreversible inhibition of cyclooxygenases leading to a longer half-life [54].

Survival of glioma patients after use of aspirin or non-aspirin NSAIDs, except for selective COX-2 inhibitors, has not much been evaluated. One pooled analysis of randomized clinical trials explored the effect of daily aspirin intake on long-term risk of death due to cancer and found a significantly reduced risk of death among brain cancer patients using daily aspirin for at least 5 years (HR = 0.31; 95% CI, 0.11–0.89) [55]. In another pooled analysis, including 1,273 patients with primary glioblastoma, anticoagulant use and anti-platelet agent use was explored in relation to patient survival and no significant associations were found [56].

In our analysis, use of aspirin but not use of other NSAIDs was associated with better overall survival in patients with WHO grade III glioma, but not in the larger group of patients with WHO grade IV glioma. Given the absence of relations between use of other NSAIDs and glioma survival, an additional COX-independent mechanism of aspirin appears likely. Patients with malignancies, including gliomas, harbour an increased risk of venous or arterial embolisms, such as deep vein thrombosis, pulmonary embolism or stroke (reviewed in [57] and [58]). Use of aspirin has little effect on venous thromboembolism [59], but as platelet aggregation inhibitor, it reduces the risk of arterial embolism, such as stroke. Secondary prevention of stroke is mostly performed with aspirin and statins [60] and use of aspirin may therefore reduce the risk of stroke among patients with glioma.

In addition to COX-dependent factors or possible prevention of arterial embolisms, aspirin might influence glioma survival by targeting platelets. Platelets are known to exert significant pro-metastatic and proinvasive properties on human cancers, for example by secreting important growth factors, such as transforming growth factor beta, vascular endothelial growth factor or platelet derived growth factor, thereby influencing the local host immune system, angiogenesis, proliferation and migration of tumor cells (reviewed in [61]) Use of aspirin as antiplatelet and anti-invasive agent has been evaluated in breast cancer, colorectal cancer and head and neck cancer with mostly positive [62–66], but also null results [67, 68], but so far there is few data on glioma. Interestingly, one study linked preoperative thrombocytosis to significantly shortened survival in glioma patients [69].

Reasons, why patients with WHO grade III gliomas have a strong survival benefit after treatment with aspirin whereas patients with WHO grade IV gliomas have not, are speculative. Potentially, patients with WHO grade III gliomas have a longer duration of aspirin use due to a higher life expectancy as compared to patients with WHO grade IV gliomas. However, results for WHO grade III glioma must be interpreted cautiously, since the analysis is likely underpowered.

Our study has several limitations mainly including the retrospective nature of the registry, frequently lacking data on duration and dose of treatment and low sample size in subgroups which may have led to false positive results. Except for low-dose aspirin, which was used by the broad majority of our aspirin patients, NSAIDs are often prescribed on demand and not on a regular basis, which may have caused us to underestimate NSAID use in our patient population, limiting our ability to detect potential associations. Confounding by indication may represent a major source of bias in our retrospective study. Patients who received aspirin may differ from patients who did not receive aspirin due to unknown factors of the underlying diseases rather than the medication itself. For example, headaches due to increased intracranial pressure as a sign of progressive glioma may have led to increased use of certain NSAIDs, thereby creating a spurious positive association with glioma for medications such as diclofenac [24–26], COX-2 inhibitors, metamizol, paracetamol or ibuprofen. This is supported by the fact, that non-aspirin NSAIDs were mostly prescribed after the diagnosis of glioma. We did however adjust our analysis for the clinical performance score, which should reduce bias due to severity of glioma symptoms, but was assessed at initial glioma diagnosis. We had large numbers of unknowns for some covariates, such as molecular markers or the extent of resection. However, there is no reason to assume that patients with favourable molecular markers or complete resection were more likely to receive statins or NSAIDS. We were not able to perform dose-response analyses because we frequently lacked information on the start date of the specific medication before glioma diagnosis. However, duration of drug use before glioma diagnosis may not be relevant for glioma survival. We were not able to stratify our analysis by steroid use, which may also influence glioma survival [70–72] and we were not able to take statin intolerance into account, which may influence duration of statin treatment.

Our study also has several notable strengths. Our full dataset has a considerable sample size and it contains information on important prognostic factors that were accounted for in our multivariate analyses. This is the first study to evaluate statins and specifically aspirin and non-aspirin NSAIDs in relation to glioma survival stratified by WHO grade. Our study is not prone to recall bias because the data regarding medications and diagnoses were collected based on hospital discharge letters and clinical notes from general practitioners collected in the cancer registry. Lastly, identification of high-grade glioma patients is likely not influenced by selection bias because patients were identified using a pre-existing database in our cancer registry.

In summary, we did not observe an association between use of aspirin and survival of patients with WHO grade IV glioma, but we found an indication for better survival of patients with WHO grade III glioma after use of aspirin. Further observational studies should be performed including higher sample sizes and more complete information on molecular marker status and intensity of treatment to validate our first results.

## Supporting information

S1 Table Baseline characteristics according to diclofenac use. (DOCX)

S2 Table Baseline characteristics according to ibuprofen use. (DOCX)

S3 Table Baseline characteristics according to use of selective COX-2 inhibitors. (DOCX)

S4 Table Baseline characteristics according to metamizol use. (DOCX)

S5 Table Baseline characteristics according to paracetamol use. (DOCX)
