# Supplementary material for: Use of statins or NSAIDs and survival of patients with high-grade glioma
Source: PLoS One. 2018 Dec 3;13(12):e0207858. doi: 10.1371/journal.pone.0207858 (PMC6277074; doi:10.1371/journal.pone.0207858)
Supplement: S1 Table — (DOCX) [file pone.0207858.s001.docx]

**S1 Table: Baseline characteristics according to diclofenac use.**

|  | | Diclofenac use | | | | | |
| --- | --- | --- | --- | --- | --- | --- | --- |
|  |  | Yes  (18, 1.6%) | | No  (1,075, 98.4%) | | Total  (1,093, 100%) | |
|  |  | count | % | count | % | count | % |
| Sex | Male | 13 | 72.2% | 606 | 56.4% | 619 | 56.6% |
|  | Female | 5 | 27.8% | 469 | 43.6% | 474 | 43.4% |
| Age at diagnosis | < 40 | 1 | 5.6% | 120 | 11.2% | 121 | 11.1% |
|  | 40-49 | 3 | 16.7% | 159 | 14.8% | 162 | 14.8% |
|  | 50-59 | 7 | 38.9% | 253 | 23.5% | 260 | 23.8% |
|  | 60-69 | 3 | 16.7% | 297 | 27.6% | 300 | 27.4% |
|  | 70-79 | 4 | 22.2% | 208 | 19.3% | 212 | 19.4% |
|  | > 80 | 0 | 0.0% | 38 | 3.5% | 38 | 3.5% |
| Year of diagnosis | 1998-2001 | 2 | 11.1% | 177 | 16.5% | 179 | 16.4% |
|  | 2002-2005 | 3 | 16.7% | 290 | 27.0% | 293 | 26.8% |
|  | 2006-2009 | 4 | 22.2% | 224 | 20.8% | 228 | 20.9% |
|  | 2010-2013 | 9 | 50.0% | 384 | 35.7% | 393 | 36.0% |
| WHO grade | 3 | 2 | 11.1% | 229 | 21.3% | 231 | 21.1% |
|  | 4 | 16 | 88.9% | 846 | 78.7% | 862 | 78.9% |
| MGMT-Promotor-Methylation | Methylation | 1 | 5.6% | 139 | 12.9% | 140 | 12.8% |
|  | Wildtyp | 4 | 22.2% | 141 | 13.1% | 145 | 13.3% |
|  | k.A. | 13 | 72.2% | 795 | 74.0% | 808 | 73.9% |
| IDH1 | Mutation | 0 | 0.0% | 54 | 5.0% | 54 | 4.9% |
|  | Wild type | 4 | 22.2% | 174 | 16.2% | 178 | 16.3% |
|  | ns | 14 | 77.8% | 847 | 78.8% | 861 | 78.8% |
| Karnofsky-Performance Score (class. ECOG) | 100 ECOG 0 | 1 | 5.6% | 139 | 12.9% | 140 | 12.8% |
|  | 80-90 ECOG 1 | 9 | 50.0% | 292 | 27.2% | 301 | 27.5% |
|  | 60-70 ECOG 2 | 3 | 16.7% | 160 | 14.9% | 163 | 14.9% |
|  | 40-50 ECOG 3 | 2 | 11.1% | 69 | 6.4% | 71 | 6.5% |
|  | 10-30 ECOG 4 | 0 | 0.0% | 9 | 0.8% | 9 | 0.8% |
|  | ns | 3 | 16.7% | 406 | 37.8% | 409 | 37.4% |
| Primary therapy | OP+Rad+Chemo | 10 | 55.6% | 481 | 44.7% | 491 | 44.9% |
|  | OP+Rad | 3 | 16.7% | 163 | 15.2% | 166 | 15.2% |
|  | OP+Chemo | 0 | 0.0% | 63 | 5.9% | 63 | 5.8% |
|  | OP | 0 | 0.0% | 102 | 9.5% | 102 | 9.3% |
|  | Rad+Chemo | 1 | 5.6% | 94 | 8.7% | 95 | 8.7% |
|  | Rad | 1 | 5.6% | 71 | 6.6% | 72 | 6.6% |
|  | Chemo | 1 | 5.6% | 15 | 1.4% | 16 | 1.5% |
|  | supportive/others | 2 | 11.1% | 86 | 8.0% | 88 | 8.1% |
| Extent of resection | complete | 0 | 0.0% | 39 | 3.6% | 39 | 3.6% |
|  | incomplete | 4 | 22.2% | 140 | 13.0% | 144 | 13.2% |
|  | biopsy | 2 | 11.1% | 52 | 4.8% | 54 | 4.9% |
|  | ns | 12 | 66.7% | 844 | 78.5% | 856 | 78.3% |
| BMI | < 25.0 | 5 | 27.8% | 186 | 17.3% | 191 | 17.5% |
|  | 25.0 - 29.9 | 4 | 22.2% | 186 | 17.3% | 190 | 17.4% |
|  | 30+ | 5 | 27.8% | 111 | 10.3% | 116 | 10.6% |
|  | ns | 4 | 22.2% | 592 | 55.1% | 596 | 54.5% |
| Total |  | 18 | 100% | 1,075 | 100% | 1,093 | 100% |
