# Supplementary material for: Use of statins or NSAIDs and survival of patients with high-grade glioma
Source: PLoS One. 2018 Dec 3;13(12):e0207858. doi: 10.1371/journal.pone.0207858 (PMC6277074; doi:10.1371/journal.pone.0207858)
Supplement: S2 Table — (DOCX) [file pone.0207858.s002.docx]

**S2 Table: Baseline characteristics according to ibuprofen use.**

|  | | Ibuprofen use | | | | | |
| --- | --- | --- | --- | --- | --- | --- | --- |
|  |  | Yes  (43, 3.9%) | | No  (1,050, 96.1%) | | total | |
|  |  | count | % | count | % | count | % |
| Sex | Male | 19 | 44.2% | 600 | 57.1% | 619 | 56.6% |
|  | Female | 24 | 55.8% | 450 | 42.9% | 474 | 43.4% |
| Age at diagnosis | < 40 | 4 | 9.3% | 117 | 11.1% | 121 | 11.1% |
|  | 40-49 | 5 | 11.6% | 157 | 15.0% | 162 | 14.8% |
|  | 50-59 | 14 | 32.6% | 246 | 23.4% | 260 | 23.8% |
|  | 60-69 | 3 | 7.0% | 297 | 28.3% | 300 | 27.4% |
|  | 70-79 | 13 | 30.2% | 199 | 19.0% | 212 | 19.4% |
|  | > 80 | 4 | 9.3% | 34 | 3.2% | 38 | 3.5% |
| Year of diagnosis | 1998-2001 | 3 | 7.0% | 176 | 16.8% | 179 | 16.4% |
|  | 2002-2005 | 5 | 11.6% | 288 | 27.4% | 293 | 26.8% |
|  | 2006-2009 | 4 | 9.3% | 224 | 21.3% | 228 | 20.9% |
|  | 2010-2013 | 31 | 72.1% | 362 | 34.5% | 393 | 36.0% |
| WHO grade | 3 | 8 | 18.6% | 223 | 21.2% | 231 | 21.1% |
|  | 4 | 35 | 81.4% | 827 | 78.8% | 862 | 78.9% |
| MGMT-Promotor-Methylation | Mutation | 8 | 18.6% | 132 | 12.6% | 140 | 12.8% |
|  | Wildtyp | 10 | 23.3% | 135 | 12.9% | 145 | 13.3% |
|  | k.A. | 25 | 58.1% | 783 | 74.6% | 808 | 73.9% |
| IDH1 | Mutation | 2 | 4.7% | 52 | 5.0% | 54 | 4.9% |
|  | Wild type | 15 | 34.9% | 163 | 15.5% | 178 | 16.3% |
|  | ns | 26 | 60.5% | 835 | 79.5% | 861 | 78.8% |
| Karnofsky-Performance Score (class. ECOG) | 100 ECOG 0 | 6 | 14.0% | 134 | 12.8% | 140 | 12.8% |
|  | 80-90 ECOG 1 | 13 | 30.2% | 288 | 27.4% | 301 | 27.5% |
|  | 60-70 ECOG 2 | 8 | 18.6% | 155 | 14.8% | 163 | 14.9% |
|  | 40-50 ECOG 3 | 6 | 14.0% | 65 | 6.2% | 71 | 6.5% |
|  | 10-30 ECOG 4 | 1 | 2.3% | 8 | 0.8% | 9 | 0.8% |
|  | ns | 9 | 20.9% | 400 | 38.1% | 409 | 37.4% |
| Primary therapy | OP+Rad+Chemo | 22 | 51.2% | 469 | 44.7% | 491 | 44.9% |
|  | OP+Rad | 7 | 16.3% | 159 | 15.1% | 166 | 15.2% |
|  | OP+Chemo | 2 | 4.7% | 61 | 5.8% | 63 | 5.8% |
|  | OP | 3 | 7.0% | 99 | 9.4% | 102 | 9.3% |
|  | Rad+Chemo | 4 | 9.3% | 91 | 8.7% | 95 | 8.7% |
|  | Rad | 2 | 4.7% | 70 | 6.7% | 72 | 6.6% |
|  | Chemo | 1 | 2.3% | 15 | 1.4% | 16 | 1.5% |
|  | supportive/others | 2 | 4.7% | 86 | 8.2% | 88 | 8.1% |
| Extent of resection | complete | 2 | 4.7% | 37 | 3.5% | 39 | 3.6% |
|  | incomplete | 10 | 23.3% | 134 | 12.8% | 144 | 13.2% |
|  | biopsy | 3 | 7.0% | 51 | 4.9% | 54 | 4.9% |
|  | ns | 28 | 65.1% | 828 | 78.9% | 856 | 78.3% |
| BMI | < 25.0 | 11 | 25.6% | 180 | 17.1% | 191 | 17.5% |
|  | 25.0 - 29.9 | 10 | 23.3% | 180 | 17.1% | 190 | 17.4% |
|  | 30+ | 7 | 16.3% | 109 | 10.4% | 116 | 10.6% |
|  | ns | 15 | 34.9% | 581 | 55.3% | 596 | 54.5% |
| Total |  | 43 | 100% | 1,050 | 100% | 1,093 | 100% |
