# Supplementary material for: Use of statins or NSAIDs and survival of patients with high-grade glioma
Source: PLoS One. 2018 Dec 3;13(12):e0207858. doi: 10.1371/journal.pone.0207858 (PMC6277074; doi:10.1371/journal.pone.0207858)
Supplement: S3 Table — (DOCX) [file pone.0207858.s003.docx]

**S3 Table: Baseline characteristics according to use of selective COX-2 inhibitors.**

|  | | Selective COX-2 inhibitor use | | | | | |
| --- | --- | --- | --- | --- | --- | --- | --- |
|  |  | Yes  (50, 4.6%) | | No  (1,043, 95.4%) | | Total  (1,093, 100%) | |
|  |  | count | % | count | % | count | % |
| Sex | Male | 33 | 66.0% | 586 | 56.2% | 619 | 56.6% |
|  | Female | 17 | 34.0% | 457 | 43.8% | 474 | 43.4% |
| Age at diagnosis | < 40 | 10 | 20.0% | 111 | 10.6% | 121 | 11.1% |
|  | 40-49 | 15 | 30.0% | 147 | 14.1% | 162 | 14.8% |
|  | 50-59 | 16 | 32.0% | 244 | 23.4% | 260 | 23.8% |
|  | 60-69 | 4 | 8.0% | 296 | 28.4% | 300 | 27.4% |
|  | 70-79 | 4 | 8.0% | 208 | 19.9% | 212 | 19.4% |
|  | > 80 | 1 | 2.0% | 37 | 3.5% | 38 | 3.5% |
| Year of diagnosis | 1998-2001 | 2 | 4.0% | 177 | 17.0% | 179 | 16.4% |
|  | 2002-2005 | 1 | 2.0% | 292 | 28.0% | 293 | 26.8% |
|  | 2006-2009 | 24 | 48.0% | 204 | 19.6% | 228 | 20.9% |
|  | 2010-2013 | 23 | 46.0% | 370 | 35.5% | 393 | 36.0% |
| WHO grade | 3 | 11 | 22.0% | 220 | 21.1% | 231 | 21.1% |
|  | 4 | 39 | 78.0% | 823 | 78.9% | 862 | 78.9% |
| MGMT-Promotor-Methylation | Mutation | 11 | 22.0% | 129 | 12.4% | 140 | 12.8% |
|  | Wildtyp | 26 | 52.0% | 119 | 11.4% | 145 | 13.3% |
|  | k.A. | 13 | 26.0% | 795 | 76.2% | 808 | 73.9% |
| IDH1 | Mutation | 4 | 8.0% | 50 | 4.8% | 54 | 4.9% |
|  | Wild type | 21 | 42.0% | 157 | 15.1% | 178 | 16.3% |
|  | ns | 25 | 50.0% | 836 | 80.2% | 861 | 78.8% |
| Karnofsky-Performance Score (class. ECOG) | 100 ECOG 0 | 14 | 28.0% | 126 | 12.1% | 140 | 12.8% |
|  | 80-90 ECOG 1 | 21 | 42.0% | 280 | 26.8% | 301 | 27.5% |
|  | 60-70 ECOG 2 | 5 | 10.0% | 158 | 15.1% | 163 | 14.9% |
|  | 40-50 ECOG 3 | 6 | 12.0% | 65 | 6.2% | 71 | 6.5% |
|  | 10-30 ECOG 4 | 0 | 0.0% | 9 | 0.9% | 9 | 0.8% |
|  | ns | 4 | 8.0% | 405 | 38.8% | 409 | 37.4% |
| Primary therapy | OP+Rad+Chemo | 28 | 56.0% | 463 | 44.4% | 491 | 44.9% |
|  | OP+Rad | 6 | 12.0% | 160 | 15.3% | 166 | 15.2% |
|  | OP+Chemo | 5 | 10.0% | 58 | 5.6% | 63 | 5.8% |
|  | OP | 3 | 6.0% | 99 | 9.5% | 102 | 9.3% |
|  | Rad+Chemo | 4 | 8.0% | 91 | 8.7% | 95 | 8.7% |
|  | Rad | 0 | 0.0% | 72 | 6.9% | 72 | 6.6% |
|  | Chemo | 2 | 4.0% | 14 | 1.3% | 16 | 1.5% |
|  | supportive/others | 2 | 4.0% | 86 | 8.2% | 88 | 8.1% |
| Extent of resection | complete | 5 | 10.0% | 34 | 3.3% | 39 | 3.6% |
|  | incomplete | 12 | 24.0% | 132 | 12.7% | 144 | 13.2% |
|  | biopsy | 5 | 10.0% | 49 | 4.7% | 54 | 4.9% |
|  | ns | 28 | 56.0% | 828 | 79.4% | 856 | 78.3% |
| BMI | < 25.0 | 18 | 36.0% | 173 | 16.6% | 191 | 17.5% |
|  | 25.0 - 29.9 | 14 | 28.0% | 176 | 16.9% | 190 | 17.4% |
|  | 30+ | 6 | 12.0% | 110 | 10.5% | 116 | 10.6% |
|  | ns | 12 | 24.0% | 584 | 56.0% | 596 | 54.5% |
| Total |  | 50 | 100% | 1,043 | 100% | 1,093 | 100% |
