# Supplementary material for: Use of statins or NSAIDs and survival of patients with high-grade glioma
Source: PLoS One. 2018 Dec 3;13(12):e0207858. doi: 10.1371/journal.pone.0207858 (PMC6277074; doi:10.1371/journal.pone.0207858)
Supplement: S4 Table — (DOCX) [file pone.0207858.s004.docx]

**S4 Table: Baseline characteristics according to metamizol use.**

|  | | Metamizol use | | | | | |
| --- | --- | --- | --- | --- | --- | --- | --- |
|  |  | Yes  (55, 5.0%) | | No  (1,038, 95.0%) | | Total  (1,093, 100%) | |
|  |  | count | % | count | count | % | count |
| Sex | Male | 26 | 47.3% | 593 | 57.1% | 619 | 56.6% |
|  | Female | 29 | 52.7% | 445 | 42.9% | 474 | 43.4% |
| Age at diagnosis | < 40 | 5 | 9.1% | 116 | 11.2% | 121 | 11.1% |
|  | 40-49 | 6 | 10.9% | 156 | 15.0% | 162 | 14.8% |
|  | 50-59 | 14 | 25.5% | 246 | 23.7% | 260 | 23.8% |
|  | 60-69 | 16 | 29.1% | 284 | 27.4% | 300 | 27.4% |
|  | 70-79 | 11 | 20.0% | 201 | 19.4% | 212 | 19.4% |
|  | > 80 | 3 | 5.5% | 35 | 3.4% | 38 | 3.5% |
| Year of diagnosis | 1998-2001 | 4 | 7.3% | 175 | 16.9% | 179 | 16.4% |
|  | 2002-2005 | 15 | 27.3% | 278 | 26.8% | 293 | 26.8% |
|  | 2006-2009 | 5 | 9.1% | 223 | 21.5% | 228 | 20.9% |
|  | 2010-2013 | 31 | 56.4% | 362 | 34.9% | 393 | 36.0% |
| WHO grade | 3 | 10 | 18.2% | 221 | 21.3% | 231 | 21.1% |
|  | 4 | 45 | 81.8% | 817 | 78.7% | 862 | 78.9% |
| MGMT-Promotor-Methylation | Mutation | 7 | 12.7% | 133 | 12.8% | 140 | 12.8% |
|  | Wildtyp | 10 | 18.2% | 135 | 13.0% | 145 | 13.3% |
|  | ns | 38 | 69.1% | 770 | 74.2% | 808 | 73.9% |
| IDH1 | Mutation | 2 | 3.6% | 52 | 5.0% | 54 | 4.9% |
|  | Wild type | 7 | 12.7% | 171 | 16.5% | 178 | 16.3% |
|  | ns | 46 | 83.6% | 815 | 78.5% | 861 | 78.8% |
| Karnofsky-Performance Score (class. ECOG) | 100 ECOG 0 | 10 | 18.2% | 130 | 12.5% | 140 | 12.8% |
|  | 80-90 ECOG 1 | 23 | 41.8% | 278 | 26.8% | 301 | 27.5% |
|  | 60-70 ECOG 2 | 5 | 9.1% | 158 | 15.2% | 163 | 14.9% |
|  | 40-50 ECOG 3 | 7 | 12.7% | 64 | 6.2% | 71 | 6.5% |
|  | 10-30 ECOG 4 | 3 | 5.5% | 6 | 0.6% | 9 | 0.8% |
|  | ns | 7 | 12.7% | 402 | 38.7% | 409 | 37.4% |
| Primary therapy | OP+Rad+Chemo | 27 | 49.1% | 464 | 44.7% | 491 | 44.9% |
|  | OP+Rad | 6 | 10.9% | 160 | 15.4% | 166 | 15.2% |
|  | OP+Chemo | 2 | 3.6% | 61 | 5.9% | 63 | 5.8% |
|  | OP | 3 | 5.5% | 99 | 9.5% | 102 | 9.3% |
|  | Rad+Chemo | 8 | 14.5% | 87 | 8.4% | 95 | 8.7% |
|  | Rad | 4 | 7.3% | 68 | 6.6% | 72 | 6.6% |
|  | Chemo | 1 | 1.8% | 15 | 1.4% | 16 | 1.5% |
|  | supportive/others | 4 | 7.3% | 84 | 8.1% | 88 | 8.1% |
| Extent of resection | complete | 1 | 1.8% | 38 | 3.7% | 39 | 3.6% |
|  | incomplete | 8 | 14.5% | 136 | 13.1% | 144 | 13.2% |
|  | biopsy | 3 | 5.5% | 51 | 4.9% | 54 | 4.9% |
|  | ns | 43 | 78.2% | 813 | 78.3% | 856 | 78.3% |
| BMI | < 25.0 | 7 | 12.7% | 184 | 17.7% | 191 | 17.5% |
|  | 25.0 - 29.9 | 17 | 30.9% | 173 | 16.7% | 190 | 17.4% |
|  | 30+ | 17 | 30.9% | 99 | 9.5% | 116 | 10.6% |
|  | ns | 14 | 25.5% | 582 | 56.1% | 596 | 54.5% |
| Total |  | 55 | 100% | 1,038 | 100% | 1,093 | 100% |
